# *LLGL2* Inhibits Ovarian Cancer Metastasis by Regulating Cytoskeleton Remodeling via *ACTN1*

**DOI:** 10.3390/cancers15245880

**Published:** 2023-12-18

**Authors:** Qiu-Ying Gu, Yue-Xi Liu, Jin-Long Wang, Xiao-Lan Huang, Ruo-Nan Li, Hua Linghu

**Affiliations:** Department of Gynecology, The First Affiliated Hospital of Chongqing Medical University, Chongqing 400016, China; guqiuying@stu.cqmu.edu.cn (Q.-Y.G.); liuyuexixi0704@gmail.com (Y.-X.L.); wangjinlong1701@163.com (J.-L.W.); hxl7181@163.com (X.-L.H.)

**Keywords:** *LLGL2*, ovarian cancer, tumor metastasis, F-actin, *ACTN1*

## Abstract

**Simple Summary:**

Epithelial ovarian cancer (EOC) is the fifth-leading cause of cancer-related deaths in women worldwide and the most lethal gynecologic malignancy. Seventy-five percent of patients are diagnosed at an advanced stage, accompanied by extensive pelvic and abdominal metastases, and thus have a poor prognosis. We first screened for critical genes in EOC in the GEO database. *LLGL2* was upregulated in ovarian cancer tissue, while low expression of *LLGL2* was significantly associated with a more advanced stage and a higher grade of EOC and a poorer survival of patients, implying that *LLGL2* may function as a tumor suppressor gene. Our data demonstrated that overexpression of *LLGL2* inhibited the ovarian cancer cell migration and invasion abilities in vivo and in vitro. Mechanistically, LLGL2 altered the intracellular localization and function of ACTN1 by interacting with ACTN1 and regulating cytoskeleton remodeling to inhibit the invasion and metastasis of ovarian cancer cells.

**Abstract:**

Epithelial ovarian cancer is the most lethal gynecological malignant tumor. Although debulking surgery, chemotherapy, and PARP inhibitors have greatly improved survival, the prognosis for patients with advanced EOC without HRD is still poor. *LLGL2*, as a cell polarity factor, is involved in maintaining cell polarity and asymmetric cell division. In the study of zebrafish development, *LLGL2* regulated the proliferation and migration of epidermal cells and the formation of cortical F-actin. However, the role of *LLGL2* in ovarian cancer has not been described. Our study found, through bioinformatics analysis, that low expression of *LLGL2* was significantly associated with a more advanced stage and a higher grade of EOC and a poorer survival of patients. Functional experiments that involved *LLGL2* overexpression and knockdown showed that *LLGL2* inhibited the migration and invasion abilities of ovarian cancer cells in vitro, without affecting their proliferation. LLGL2-overexpressing mice had fewer metastatic implant foci than the controls in vivo. Mechanistically, immunoprecipitation combined with mass spectrometry analysis suggested that LLGL2 regulated cytoskeletal remodeling by interacting with ACTN1. LLGL2 altered the intracellular localization and function of ACTN1 without changing its protein and mRNA levels. Collectively, we uncovered that LLGL2 impaired actin filament aggregation into bundles by interacting with ACTN1, which led to cytoskeleton remodeling and inhibition of the invasion and metastasis of ovarian cancer cells.

## 1. Introduction

Epithelial ovarian cancer (EOC) is the fifth-leading cause of cancer-related deaths in women worldwide [1]. EOC is the most lethal gynecological cancer. Owing to the lack of specific symptoms and screening methods, approximately 75% of patients are diagnosed at an advanced stage. The 5-year survival rate at this late stage is below 30%, contributing to the high death-to-incidence rate [2]. EOC is classified into high-grade serous ovarian carcinoma (HGSOC), low-grade serous ovarian carcinoma (LGSOC), mucinous carcinoma (MC), endometrioid carcinoma (EC), and ovarian clear cell carcinoma (OCCC). OCCC is frequently diagnosed at an early stage. However, when diagnosed at an advanced stage, it shows a more severe and poor prognosis than other subtypes [3]. Thus, studying the molecular mechanisms of ovarian cancer cell metastasis and seeking new therapeutic targets are of great significance in improving the long-term survival of patients.

Every cell has an inherent ability to asymmetrically distribute cell membrane and intracellular components. This asymmetry is used to perform directional functions, such as an interaction with the environment or migration toward cues, vectorial transport, and absorption of molecules from the extracellular space. Such an asymmetric feature is referred to as “cell polarity” and is involved in multiple cellular processes, including cell proliferation, differentiation, asymmetric cell division, cell migration, tissue morphogenesis, and tumor formation [4,5]. LLGL Scribble cell polarity complex component 2 (*LLGL2*) is a mammalian homolog of *Drosophila* LGL. An important paralog of *LLGL2* is *LLGL1*. Lethal giant larvae (*LGL*) were first discovered in *Drosophila* and are a component of the Scribble polarity complex. The Scribble polarity complex comprises SCRIB, DLG, and LGL, which regulate the maintenance of epithelial apical–basal polarity (ABP) and asymmetric cell division [6]. The apical–basal polarity is essential for the formation and function of epithelial cells. Early studies indicated that the loss of normal function of *Drosophila* polar proteins could induce the occurrence and progression of cancer-like phenotypes [5]. Loss-of-function mutations in genes such as *DLG*, *LGL*, and *SCRIB* in *Drosophila* lead to the loss of epithelial cell polarity and to uncontrolled proliferation, properties usually associated with neoplastic tumor suppressor genes [5,7,8]. The LGL protein interacts with atypical protein kinase C (aPKC), which phosphorylates the serine-rich loop in LGL, thereby regulating its interactions with other polar proteins and lipid membranes [9,10,11,12]. In zebrafish epidermal development, the LGL2 function is necessary for hemidesmosome granule formation and the maintenance of tissue integrity in the developing basal epidermis, and the deletion of LGL2 leads to hyperproliferation and migration [13,14]. In zebrafish epidermal microridge elongation, antagonistic interactions between aPKC and LGL control the microridge length. aPKC regulates the LGL levels in the apical cortex to inhibit actin polymerization-dependent microridge elongation. LGL regulates cortical F-actin formation and F-actin assembly in the basolateral domain [15]. In mammalian cancer development, the function of polar proteins is still unclear. *LLGL2* expression is reduced in colorectal and breast cancers, and overexpression of zinc finger E box-binding homologous protein 1 (ZEB1) inhibits *LLGL2* expression, leading to the loss of epithelial cell polarity and increased metastasis [16]. However, cell polarity proteins are not always lost in cancer, and polarity genes have been found to be amplified and overexpressed in a variety of cancers [4]. *LLGL2* is highly expressed in estrogen receptor-positive (ER+) breast cancer, and *LLGL2* regulates the cell surface levels of the leucine transporter protein SLC7A5 through the formation of a trimeric complex with SLC7A5 and the membrane fusion regulator YKT6 to promote leucine uptake and cell proliferation; *LLGL2* is also involved in tamoxifen resistance [17]. *LLGL2*, as a scaffold protein for protein–protein and protein–RNA interactions, plays an important role in tumor progression [17,18]. Cell polarity proteins may play more complex roles in mammals and cancer development than their classical cell polarity functions reported in model organisms.

Our study found that *LLGL2* was upregulated in ovarian cancer tissues, but its levels negatively correlated with malignant progression and the poor prognosis of ovarian cancer. We found that *LLGL2* altered the intracellular localization and function of *ACTN1* by interacting with *ACTN1* and regulating cytoskeleton remodeling to inhibit the invasion and metastasis of ovarian cancer cells.

## 2. Materials and Methods

### 2.1. Bioinformatics Analysis

GSE65986 and GSE6008 gene expression profiles were obtained from the GEO database [19,20,21]. Differentially expressed genes (DEGs) between the good and poor prognosis group samples were identified with the R limma package. A value of |log2 FoldChange| > 1 and a *p* value < 0.05 were considered threshold values for DEGs in GSE65986. Batch survival analysis was conducted using the log-rank test method with the R survival and survminer packages. GEO2R (https://www.ncbi.nlm.nih.gov/geo/geo2r/ (accessed on 10 January 2022)) is a data processing tool for GEO. A value of |log2 FoldChange| > 1 and a *p* value < 0.05 were considered the cutoff criteria for DEGs in GSE6008. Key genes were identified by using the intersection of the two sets in a Venn diagram. Metascape (https://metascape.org (accessed on 10 February 2022)) [22] was used to identify and visualize the top statistically enriched terms of the 42 key DEGs. CSIOVDB (http://csiovdb.mc.ntu.edu.tw/CSIOVDB.html (accessed on 10 February 2022)) [23] is a transcriptomic microarray database of 3431 human ovarian cancers, including cancers of the fallopian tube, peritoneum, metastasis to the ovary from other sites, and carcinoma of the ovary, with major ovarian cancer histologies (clear cell, endometrioid, mucinous, low-grade serous, and serous). We used CSIOVDB for clinical pathological analysis and survival analysis. The Gene Expression Profiling Interactive Analysis 2 (GEPIA2) database (http://gepia2.cancer-pku.cn/ (accessed on 10 February 2022)) is an upgraded web server for large-scale expression profiling and interactive analysis [24]. GEPIA2 was used to perform a differential gene expression analysis of tumor and normal tissues. cBioPortal (www.cbioportal.org (accessed on 10 February 2022)), a comprehensive web resource, allows for the exploration, visualization, and analysis of multidimensional cancer genomics data [25,26]. TIMER2.0 (http://timer.cistrome.org/ (accessed on 10 February 2022)) was used to explore the associations between gene expression and tumor features in TCGA. The statistical significance computed by the Wilcoxon test is annotated by the number of stars (*: *p* value < 0.05; **: *p* value < 0.01; ***: *p* value < 0.001). The Kaplan–Meier Plotter (http://kmplot.com (accessed on 10 February 2022)) was used to perform prognostic analysis.

### 2.2. Cell Lines

The human ovarian clear cell adenocarcinoma ES-2 (RRID: CVCL_3509) and human high-grade ovarian serous adenocarcinoma OVCAR-3 (RRID: CVCL_0465) cell lines were originally obtained from American Type Culture Collection (ATCC) and cultured in RPMI-1640 medium supplemented with 10% fetal bovine serum (FBS) and 100 U/mL penicillin–streptomycin (C0222; Beyotime, Shanghai, China). All cells were cultured in a humidified incubator at 37 °C and a 5% CO_2_ atmosphere. All cells were identified by short-tandem-repeat profiling and had no mycoplasma contamination.

### 2.3. Transfections and Lentiviral Infection

Lentiviral vectors for overexpression of *LLGL2* were obtained from Genechem Co., Ltd. (Shanghai, China). Transfection was performed according to the manufacturer’s protocol. Stably transfected cells were selected using puromycin. The small interfering RNAs for *LLGL2* were obtained from Tsingke Biology Co., Ltd. (Beijing, China). Transfection was performed using Lipofectamine 2000 (no.11668019, Invitrogen, Carlsbad, CA, USA) according to the manufacturer’s protocol. The transfection efficiency was determined by RT–qPCR and Western blot analyses.

### 2.4. Western Blot Analysis

Whole-cell lysates obtained using RIPA lysis buffer were added to loading buffer and then boiled. Samples were separated by 8%–10% SDS–PAGE, and proteins were transferred to polyvinylidene fluoride membranes (Millipore, Billerica, MA, USA). Blots were blocked with 5% milk and then incubated with antibodies against *LLGL2* (1:100, no. sc-376857, Santa Cruz Biotechnology, Santa Cruz, CA, USA), *ACTN1* (1:1000, no. ER1803-60, Huabio, Hangzhou, China), and GAPDH (1:5000, no. 10494-1-AP, Proteintech, Wuhan, China) at 4 °C overnight. An HRP-coupled anti-rabbit or anti-mouse secondary antibody (Boster, Wuhan, China) was used at a 1:10,000 dilution ratio, and blots were incubated for 2 h at room temperature. Then, we applied a chemiluminescence reagent (ECL, Meilunbio, Dalian, China) to the membranes and exposed them to a Fusion imaging system (FUSION FX7 EDGE V.070, Vilber, COLLEGIEN, France). All experiments were repeated at least three times, and representative data are shown.

### 2.5. Transwell and Wound Healing Assays

Cells were cultured in a 6-well culture plate until 90% confluency was reached and then were scraped with a 10 µL sterile pipette tip. At 0, 24, and 48 h, the scratched area was observed under a light microscope (Olympus, Japan). For the migration assay, 0.5–1 × 10^4^ cells were plated into each upper chamber of a 24-well Transwell insert with 8 μm pores (Corning, NY, USA) and cultured in 200 μL of a serum-free medium. The bottom chambers were filled with 700 μL of RPMI-1640 medium supplemented with 10% FBS. After 10–12 h of incubation, the cells were fixed with 4% paraformaldehyde and stained with crystal violet. Images of three or four randomly selected fields of view were captured by microscopy for examination. Similarly, to determine the invasion capacity of cells, Matrigel™ (no. 356,234, Corning, NY, USA) was mixed with a serum-free RPMI-1640 medium at a ratio of 1:39, and this mixture was added to a Transwell^®^ top chamber. After 27–36 h of incubation, cells were fixed. The other steps were similar to those of the migration assay.

### 2.6. Phalloidin Staining

Cells were fixed with 4% paraformaldehyde for 20 min, permeabilized with 0.1% Triton X-100 for 15 min, washed with PBS three times, and stained with a fluorescent phalloidin solution (no. C2201S, Beyotime, China) according to the manufacturer’s instructions for 1 h at room temperature. Actin-Tracker was diluted with PBS containing 5% BSA and 0.1% Triton X-100 at a 1:100 ratio. Then, the cells were washed with PBS and photographed under a confocal microscope (LSM 800, Zeiss, Jena, Germany).

### 2.7. Coimmunoprecipitation (Co-IP) and Liquid Chromatography–Mass Spectrometry

Total protein from cells stably transfected with the LLGL2 expression vector or an empty vector was extracted in a weak lysis buffer (Cell lysis buffer for Western and IP, No.P0013, Beyotime, China) supplemented with a proteinase inhibitor. Cells in one T175 bottle were lysed with 500 µL of the lysis solution and treated with ultrasound to obtain the total protein. For Co-IP, 800 μg of the protein extract was incubated overnight at 4 °C on a rotator with a primary anti-LLGL2 antibody (1 μg per 100 μg of protein; no. sc-376857; Santa Cruz Biotechnology). Then, immune complexes were precipitated with protein A/G mix magnetic beads (20 μL, MCE) for 4 h at 4 °C. The mixture was then magnetically separated and washed three times with PBST. Samples were resuspended in 20 μL of gel loading buffer, and Western blotting was performed as described above. For mass spectrometry analysis, SDS–PAGE was performed, and the gel was stained with Coomassie brilliant blue R250. We cut the whole gel strip (>10 kDa) for LC–MS (Novogene, Beijing, China). The in-gel digestion and next steps were operated by the Novogene company. The protocol of in-gel digestion was as follows: 100 μL with 100 mM TEAB, 1 μg trypsin and 1/300 CaCl_2_ were added into gel, proteins were digested overnight at 37 °C. After centrifugation at low speed, the supernatant was collected, 200 µL of acetonitrile was added, put under a vortex, and mixed. And the supernatant was extracted in 100 μL of 0.1% formic acid (FA). The supernatant was combined and centrifuged at 12,000× *g* for 5 min at room temperature and then lyophilized. The powder was dissolved and mixed in 0.1% of formic acid. The supernatant was slowly loaded to the C18 desalting column, washed with washing solution (0.1% formic acid, 3% acetonitrile) three times, then eluted twice by some elution buffer (0.1% formic acid, 70% acetonitrile). The eluents were collected and lyophilized. UHPLC–MS/MS analyses were performed using an EASY-nLCTM 1200 UHPLC system (Thermo Fisher, Karlsruhe, Germany) coupled with a Q ExactiveTM HF-X mass spectrometer (Thermo Fisher, Germany).

### 2.8. Immunofluorescence Staining

Cells were fixed with 4% paraformaldehyde and then permeabilized with 0.1% Triton X-100. After washing with PBS three times, the sample was blocked with goat serum albumin (Boster, China) for 30 min at room temperature. Primary antibodies were diluted with 1× PBS, and the cells were incubated with a primary antibody solution overnight at 4 °C. Antibodies were diluted as follows: anti-LLGL2 (1:100; no. sc-376857, Santa Cruz Biotechnology, Santa Cruz, CA, USA) and anti-ACTN1 (1:200; no. ER1803-60, Huabio, Hangzhou, China). After three washes with 1× PBS, the cells were incubated with Alexa Fluor 647 (1:400; Beyotime, no. A047) and DyLight 488 (1:400; Abbkine, Wuhan, China, no. A23220) for an hour at room temperature. The nuclei were stained with DAPI for 5 min. Images were captured using confocal microscopy (LSM 800, Zeiss).

### 2.9. Tumor Xenograft Models

The Animal Care and Use Committee of Chongqing Medical University approved all animal protocols. Athymic female nude mice (BALB/c nu/nu, aged four weeks) were purchased from Vital River (Beijing, China) and maintained at our institution’s pathogen-free facility. Human ovarian cancer cells were collected and inoculated into the peritoneal cavities of 6-week-old mice (4 × 10^6^ cells suspended in 200 μL of PBS per mouse; *n* = 5 per group). One month after the injection, the animals were euthanized, and metastases in the peritoneal cavity were assessed.

### 2.10. Immunohistochemistry (IHC)

IHC staining was performed on 6 μm sections of paraffin-embedded tumor samples to determine the LLGL2 expression level. PBS treatment was used as a negative control. All sections were deparaffinized in xylene, dehydrated in alcohol, incubated with hydrogen peroxide, blocked with goat serum, and then incubated with an anti-LLGL2 antibody (1:100; no. sc-376857, Santa Cruz Biotechnology, Santa Cruz, CA, USA). All sections were exposed to biotin-conjugated goat anti-rabbit IgG (Bei Jing Zhong Shan-Golden Bridge Technology Co., Ltd., Beijing, China). Images were captured using Carl Zeiss microscope (Axio Imager A2, Carl Zeiss, Jena, Germany).

### 2.11. Statistical Analysis

All data were obtained from at least three repetitions of each experiment. GraphPad Prism 8.0 (GraphPad Software, San Diego, CA, USA) was used to analyze the data. All data are shown as the mean ± SD. Student’s *t* test was used to analyze the differences between two groups with a normal distribution. The Mann–Whitney test was applied to compare ranks in nonparametric data. One-way ANOVA was used to compare three or more groups. Kaplan–Meier plots were applied to analyze the patient survival time. Probability values less than 0.05 were considered statistically significant.

## 3. Results

### 3.1. Identification of LLGL2 as a Key Gene in Ovarian Cancer

**Figure 1:**
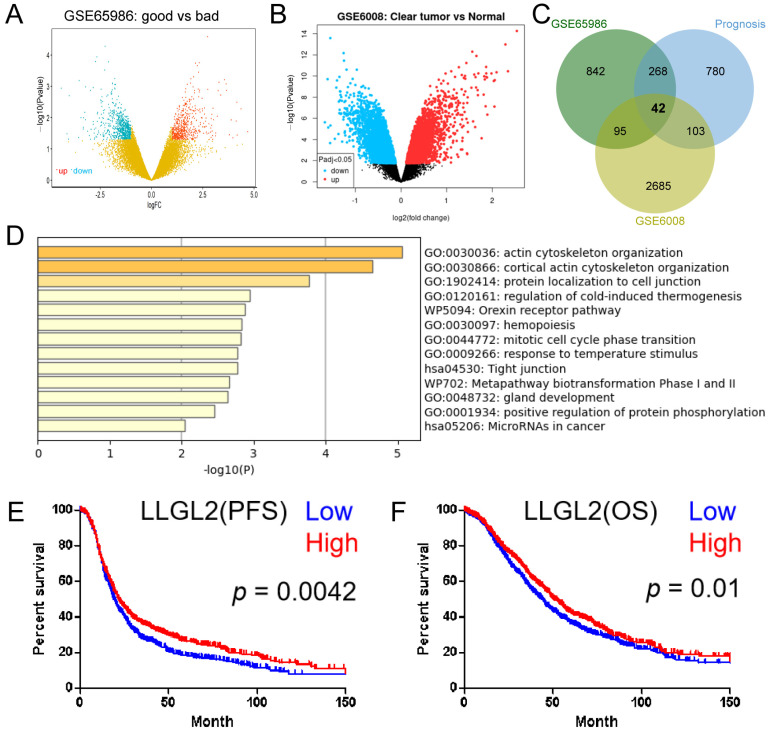
Identification of *LLGL2* as a key gene in ovarian cancer. (**A**,**B**) Volcano plots of DEGs in GSE65986 and GSE6008. The red dots indicate upregulated DEGs, and the blue dots indicate downregulated DEGs. (**C**) Venn diagram of the overlapping DEGs in GSE65986 and GSE6008. (**D**) Enrichment analysis of 42 DEGs using Metascape. (**E**,**F**) Progression-free survival (PFS) (**E**) and overall survival (OS) (**F**) curves of *LLGL2* in patients with ovarian cancer by using CSIOVDB.

To identify genes that play a key role in the survival of patients with ovarian clear cell carcinoma, we found two OCCC datasets in the GEO database. Firstly, in GSE65986, the patients were divided into two groups based on their prognostic information (Supplementary Table S1). With |log2 FC| > 1 and a *p* value < 0.05 as the critical values, 1247 DEGs were identified in GSE65986 using the R limma package (Figure 1A). We used GEO2R to extract 2925 DEGs between OCCC and normal tissues from GSE6008 (*p* < 0.05) (Figure 1B). Then, 1193 genes associated with survival (*p* value < 0.05) were screened by univariate survival analysis in GSE65986. Finally, 42 overlapping DEGs were discovered in the above three gene sets using a Venn diagram (Figure 1C). To understand the biological functions and pathways of the overlapping DEGs, a functional enrichment analysis was performed using Metascape (https://metascape.org (accessed on 10 February 2022)). The results showed that the DEGs were enriched in actin cytoskeleton organization, cortical actin cytoskeleton organization, protein localization to cell junction, hemopoiesis, and mitotic cell cycle phase transition (Figure 1D). According to the enrichment analysis results, the most enriched terms of DEGs were related to actin cytoskeleton organization, which is associated with tumor metastasis [27]. The genes enriched in the first two columns, GO: 0030036 and GO: 0030866, are shown in Table S2.

To identify significant genes in ovarian cancer, we analyzed a large sample database. Further screening in the ovarian cancer database CSIOVDB [23] revealed that only *LLGL2* showed significant differences in OS and PFS (Figure 1E,F). There were no statistically significant differences in other genes (ADD2, ACTN4, HIP1R, EPB41L1, CLSTN1, PLCL1, BIK, CIT, NCKAP1, and CDK2AP2; Supplementary Figure S1). Moreover, multivariate Cox regression analysis showed that *LLGL2* was still an independent prognostic protective factor for PFS (CSIOVDB, HR = 0.835, *p* = 0.04). Therefore, *LLGL2* may play a key role in the occurrence and development of ovarian cancer.

### 3.2. LLGL2 Correlated with Tumor Progression and a Better Prognosis in Ovarian Cancer

**Figure 2:**
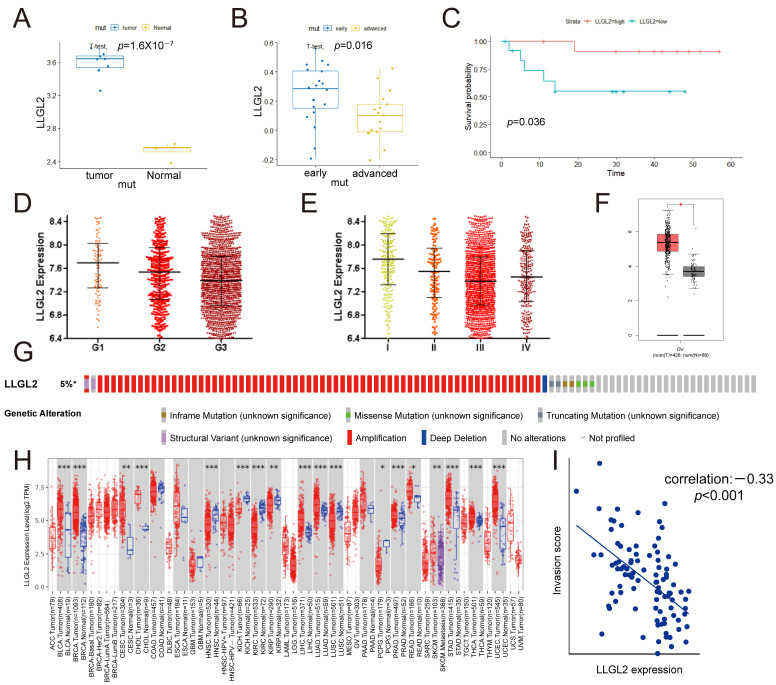
High *LLGL2* expression in ovarian cancer was inversely correlated with malignant progression and a poor prognosis. (**A**) *LLGL2* mRNA expression levels in OCCC and normal tissues in GSE6008. (**B**) *LLGL2* mRNA expression levels in early-stage and advanced-stage OCCC in GSE65986. (**C**) PFS curves of *LLGL2* in patients with OCCC from GSE65986 (*n* = 25). (**D**) *LLGL2* was negatively correlated with high grades of ovarian cancer (G1 vs. G3, *p* < 0.0001; CSIOVDB). (**E**) *LLGL2* was negatively correlated with advanced-stage ovarian cancer (I vs. III, *p* < 0.0001; CSIOVDB). (**F**) *LLGL2* expression in SOC tumors. (**G**) Genetic alteration of *LLGL2* in ovarian cancer. (**H**) Differential expression of *LLGL2* between tumor and normal tissues in TCGA pancancer chips. (**I**) *LLGL2* was negatively correlated with the invasion score from cancerSEA (*p* < 0.001). * *p* < 0.05, ** *p* < 0.01, *** *p* < 0.001.

In GSE6008, we discovered that the expression levels of *LLGL2* mRNA were considerably higher in OCCC tissues than in normal tissues (*p* = 1.6 × 10^−7^; Figure 2A). In GSE65986, we discovered that the expression of *LLGL2* was downregulated in advanced OCCC compared with early OCCC (Figure 2B), and a high expression level of *LLGL2* in OCCC was associated with a longer PFS (Figure 2C). In CSIOVDB, a transcriptomic microarray database of 3431 human ovarian cancers, low expression of *LLGL2* was correlated with an advanced tumor stage and high grades of ovarian cancer (I vs. III, *p* < 0.0001; G1 vs. G3, *p* < 0.0001; Figure 2D,E). The expression level of *LLGL2* (but not *LLGL1*) mRNA in ovarian serous cystadenocarcinoma was significantly higher than that in normal tissues (Figure 2F and Figure S2B). Moreover, the expression level of LLGL1 did not exhibit an equal prognostic value (Figure S2C). In addition, the *LLGL2* gene was amplified in 5% of patients with ovarian cancer, and this amplification correlated with the mRNA levels (Figure 2G and Figure S2A). *LLGL2* was aberrantly expressed in various cancer tissues, suggesting that it may be involved in tumor progression (Figure 2H).

Collectively, these results suggested that *LLGL2* was upregulated in ovarian cancer tissues but negatively correlated with malignant progression and a poor prognosis of ovarian cancer. *LLGL2* expression was further reduced in advanced-stage and higher-grade tumors, suggesting a potential role of *LLGL2* loss in metastasis (Figure 2B,D,E,I). *LLGL2* may play a role as a tumor suppressor gene in ovarian cancer. Thus, the roles of *LLGL2* in tumorigenesis and subsequent tumor progression may be separated.

### 3.3. LLGL2 Inhibited Ovarian Cancer Cell Migration and Invasion In Vitro

**Figure 3:**
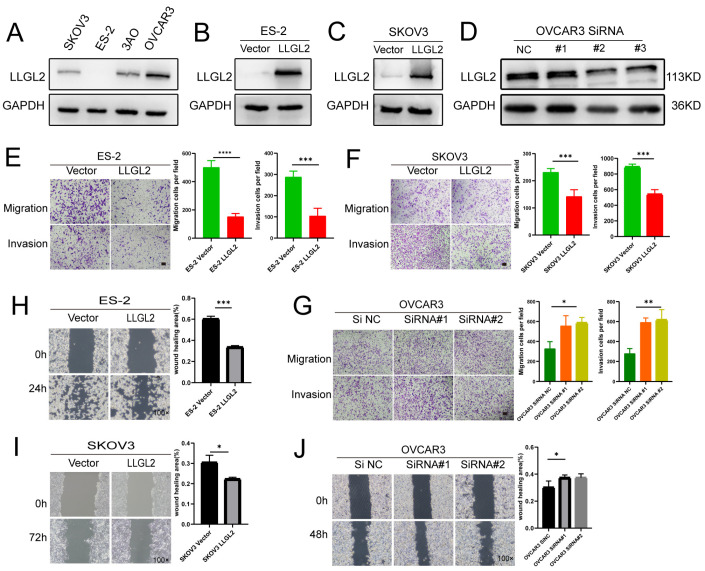
*LLGL2* inhibited the migration and invasion of ovarian cancer cells. (**A**) Relative protein expression levels of *LLGL2* in ovarian cancer cell lines. (**B**) Efficiency of lentiviral *LLGL2* overexpression in OCCC ES-2 cells was tested by Western blotting. (**C**) Efficiency of lentiviral *LLGL2* overexpression in SOC cells SKOV3 was tested by Western blotting. (**D**) Efficiency of *LLGL2* silencing in SOC cells OVCAR3 by small interfering RNA was tested by Western blotting. (**E**,**F**) Transwell migration and invasion of control (Vector) and *LLGL2*-overexpressing ES-2 and SKOV3 cells. Scale bars, 100 μm. (**G**) Transwell migration and invasion of control (NC) and *LLGL2* knockdown OVCAR3 cells. Scale bars, 100 μm. (**H**,**I**) Wound healing assay of control (Vector) and *LLGL2*-overexpressing ES-2 and SKOV3 cells. (**J**) Wound healing assay of control (NC) and *LLGL2* knockdown OVCAR3 cells. * *p* < 0.05, ** *p* < 0.01, *** *p* < 0.001, **** *p* < 0.0001. The uncropped bolts are shown in Supplementary Materials.

Firstly, we characterized the relative mRNA and protein expression levels of *LLGL2* in the ovarian cancer cell lines. Based on the RT–qPCR and Western blotting results, we found that *LLGL2* was highly expressed in OVCAR3 (SOC) cells, and its expression was low in SKOV3 (SOC) and ES-2 (OCCC) cells (Figure 3A and Figure S3A). To test its function, we then overexpressed *LLGL2* via lentivirus transfection in ES-2 and SKOV3 cells (Figure 3B,C and Figure S3B,C). At the same time, we knocked down *LLGL2* using siRNA in OVCAR3 cells (Figure 3D and Figure S3D).

We found that *LLGL2* overexpression significantly inhibited the ES-2 and SKOV3 cell passage through 8 μm pores in the Transwell migration and invasion assays (Figure 3E,F). At the same time, the knockdown of *LLGL2* promoted the confined migration and invasion capacities of OVCAR3 cells (Figure 3G). Consistently, we found that *LLGL2* overexpression could significantly slow the wound healing of ES-2 cells (Figure 3H) and SKOV3 cells (Figure 3I). At the same time, the knockdown of *LLGL2* inhibited the unconfined migration (wound healing) of OVCAR3 cells (Figure 3J). However, we did not find significant changes in cell proliferation (Figure S3E–G) or colony formation (Figure S3H,I). These results showed that *LLGL2* inhibited the migration and invasion of ovarian cancer cells in vitro without affecting cell proliferation.

### 3.4. LLGL2 Suppressed the Dissemination of Ovarian Cancer Cells In Vivo

Metastasis from epithelial ovarian cancer can occur via the transcoelomic, hematogeneous, or lymphatic route. Of these, transcoelomic metastasis is the most common and is responsible for the greatest morbidity and mortality in women with this disease [28].

**Figure 4:**
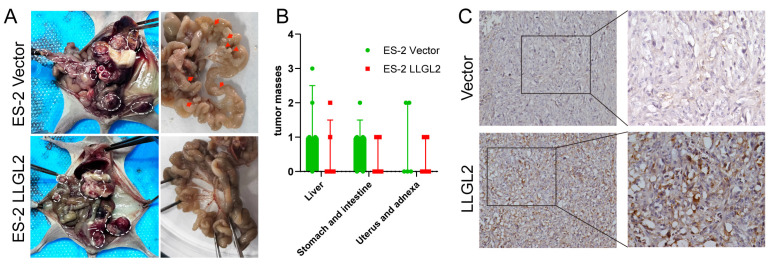
*LLGL2* suppressed the dissemination of ovarian cancer in vivo. (**A**) Representative images of tumor formation in the peritoneal cavity of mice injected with the vector and with *LLGL2*-overexpressing ES-2 cells. (**B**) Numbers of tumor masses in the abdominal cavity according to the target organ. (**C**) Tumors from each group were immunohistochemically tested for LLGL2 expression. Original magnifications: left, ×200; right, ×400.

To study the role of *LLGL2* in inhibiting metastases in vivo, control (vector) and *LLGL2*-overexpressing ES-2 cells were intraperitoneally injected into nude mice (five mice per group). As shown in Figure 4A, overt metastatic implants were observed on the liver, stomach, spleen, intestine, uterus, and adnexa in mice in the cachectic state. In the control group, the surface of the small intestine was covered with miliary nodules, while in the *LLGL2*-overexpressing group, there were only two local nodules (diameter >1 mm). The *LLGL2*-overexpressing mice had fewer metastatic implant foci than the controls, although there was no significant difference (Figure 4B). In addition, the histological results of xenograft tumors showed that the vector group had a diffuse low expression of LLGL2, while the LLGL2-overexpressing group had a significant increase in LLGL2 expression, and we found its nonuniform enhancement along the cell membrane (Figure 4C).

### 3.5. LLGL2 Interacted with ACTN1 and Impaired Actin Filament Aggregation into Bundles

Epithelial–mesenchymal transition (EMT) is involved in tumor cell migration, invasion, and metastasis [29,30]. It is generally thought that epithelial cell polarity is lost during EMT, with a loss of the cuboidal or columnar morphology of epithelial cells and a gain of the mesenchymal-like elongated morphology. Although mesenchymal cells no longer possess the apical–basal polarity, they continue to express polarity proteins. In mesenchymal cells, polarity proteins are rewired to regulate front–rear polarization processes and directional cell migration [4,31]. The EMT-related transcription factors ZEB1, snail, and SOX2 can inhibit the expression of multiple epithelial polarity genes, affect the function of polarity complexes, and induce EMT [16,32,33,34]. One of the hallmarks of EMT is the downregulation of E-cadherin and the upregulation of vimentin. To reveal how *LLGL2* regulates ovarian cancer cell migration and invasion, we first investigated whether *LLGL2* affected the EMT status. As shown in Figure S4, although the *LLGL2* expression levels were significantly correlated with the EMT scores, *LLGL2* did not change the expression levels of the EMT markers (CDH1 and VIM) or cell junction genes (TJP1 and OCLN) in cells. The inhibitory effect of *LLGL2* on ovarian cancer metastasis seems to be independent of EMT.

**Figure 5:**
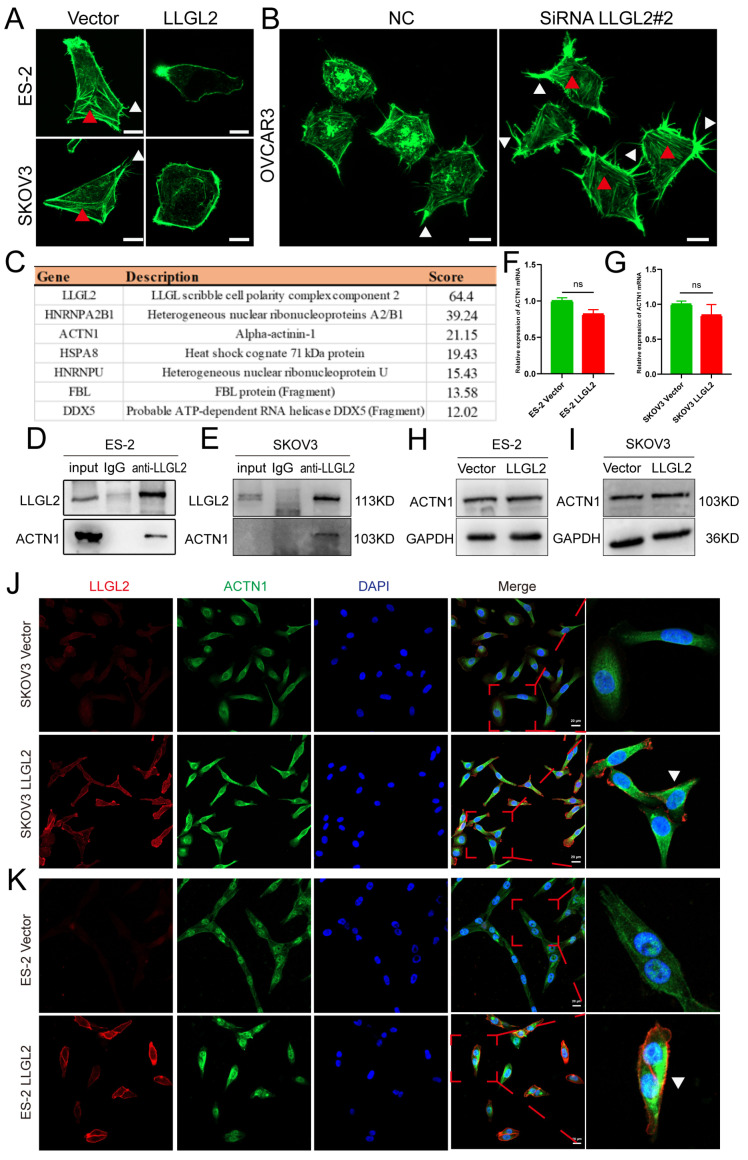
*LLGL2* interacted with *ACTN1* and impaired actin filament aggregation into bundles. (**A**) Representative images of phalloidin staining of control (Vector) and *LLGL2*-overexpressing ES-2 and SKOV3 cells. Scale bar: 10 µm. (**B**) Representative phalloidin staining images of control (NC) and *LLGL2* knockdown OVCAR3 cells. Scale bar: 10 µm. (Red triangles indicate stress fibers within the cell, and white triangles indicate filopodia around the cell). (**C**) List of the *LLGL2*-binding proteins identified by Co-IP/MS analysis. (**D**,**E**) Coimmunoprecipitation showed the binding between *LLGL2* and *ACTN1* in *LLGL2*-overexpressing ES-2 and SKOV3 cells. (**F**,**G**) RT–qPCR results showing stable *ACTN1* mRNA expression in *LLGL2*-overexpressing ES-2 and SKOV3 cells. (**H**,**I**) Immunoblots for *ACTN1* levels in control (vector) and *LLGL2*-overexpressing ES-2 and SKOV3 cells. (**J**,**K**) *ACTN1* is polarized on one side of *LLGL2*-overexpressing cells (triangles). Scale bars, 20 μm. The uncropped bolts are shown in Supplementary Materials.

To determine whether the metastasis-suppressive effect of *LLGL2* depends on actin cytoskeleton organization, we performed phalloidin staining. The results showed that *LLGL2* overexpression significantly reduced the numbers of intracellular stress fibers and filopodia compared with those in the empty vector control group (Figure 5A). In contrast, the knockdown of *LLGL2* increased the formation of intracellular stress fibers, the number of filopodia, and the filopodium length compared with those in the negative control (Figure 5B). *LLGL2* can function as a scaffolding molecule for protein interactions in biological processes. To discover possible interacting molecules for *LLGL2* in ovarian cancer, we obtained LLGL2-bound protein complexes by coimmunoprecipitation with a monoclonal antibody specific for *LLGL2* in *LLGL2*-overexpressing ES-2 cells. Subsequent mass spectrometry analysis showed that LLGL2 could specifically bind to α-actinin-1 (Figure 5C), an actin-binding protein. α-actinin-1 family proteins are ubiquitously expressed, and to date, at least four human α-actinin genes have been described, of which α-actinins 1 and 4 are ubiquitously expressed in nonmuscle cells. α-Actinin-1 consists of three conserved domains, an N-terminal actin-binding module, a central region consisting of four spectrin-like repeats, and a C-terminal calmodulin-binding domain. α-Actinin-1 directly connects actin filaments through the N-terminal actin-binding domain. ACTN1 crosslinks actin filaments into actin bundles and networks. Alpha-actinin-1 phosphorylation modulates pressure-induced colon cancer cell adhesion [35]. ACTN1 has been reported to be phosphorylated on its actin-binding domain by the focal adhesion kinase, and phosphorylation negatively regulates the binding of ACTN1 to actin [35]. It has been found that the cleavage of lymphocyte function-associated antigen-1 (LFA-1) by cathepsin X promotes ACTN1 binding to LFA-1, leading to the enhanced migration of T cells [36]. Cathepsin X induces the translocation of α-actinin-1. A-Actinin-1 was found to be predominantly localized to part of the cell membrane, presumably forming the leading edge of cathepsin X-overexpressing T cells. However, ACTN1 in WT T cells is primarily dispersed in the cytoplasm and the nucleus.

In our study, coimmunoprecipitation experiments confirmed that the LLGL2 and ACTN1 proteins could bind together in ES-2 and SKOV3 cells (Figure 5D,E). Nevertheless, LLGL2 did not significantly change the mRNA and protein levels of *ACTN1* (Figure 5F–I). Interestingly, *LLGL2* induced the translocation of α-actinin-1. Firstl**y**, when ES-2 and SKOV3 cells were immunostained for *LLGL2*, the fluorescence in the control group (vector) was weak and diffuse and was evenly distributed between the cytoplasm and the membrane. However, with a stronger fluorescence following *LLGL2* overexpression, *LLGL2* was distributed more heavily along the cell membrane. In addition, α-actinin-1 was enriched on one side of the cell in *LLGL2*-overexpressing cells, while in the vector cells, α-actinin-1 was mainly dispersed in the cytoplasm and the nucleus (Figure 5J,K). Thus, these results demonstrated that *LLGL2* altered the intracellular localization and function of *ACTN1* by interacting with *ACTN1* and impaired actin filament aggregation into bundles.

## 4. Discussion

Epithelial ovarian cancer (EOC) is the most lethal gynecologic malignancy, and 70–80% of SOC patients are diagnosed at an advanced stage. Although the use of poly (ADP-ribose) polymerase inhibitors (PARPi) has greatly improved the survival of ovarian cancer patients in recent years, only half of the patients with homologous recombination defects (HRDs) benefit greatly, and most patients will develop primary or secondary resistance to the drug. Therefore, it is necessary to explore the mechanisms of invasion and metastasis of ovarian cancer and find new therapeutic targets. Ovarian cancer has significant heterogeneity, with clear cell ovarian cancer being more likely to be detected in the early stage. Early diagnosis usually leads to a good prognosis, while advanced patients with clear cell ovarian cancer may have a worse prognosis than those with other histological types.

In this study, we first conducted a preliminary screening via bioinformatics analysis. In two ovarian clear cell carcinoma datasets, we screened for differentially expressed genes between cancer and normal ovarian tissues and for differentially expressed genes related to prognosis. Through functional enrichment and survival curve analysis, we screened for the key genes closely related to the survival of patients with ovarian cancer. We found that *LLGL2* was differentially expressed in ovarian cancer tissues. *LLGL2* was upregulated in ovarian cancer tissues, but its levels were negatively correlated with malignant progression and the poor prognosis of ovarian cancer. Low expression of *LLGL2* was significantly associated with an advanced stage and a higher grade of ovarian cancer and a poorer survival of patients. Thus, *LLGL2* may function as a tumor suppressor gene in the context of ovarian cancer spreading. The roles of LLGL2 in tumorigenesis and subsequent tumor progression may be separated. Next, *LLGL2* overexpression and knockdown experiments showed that *LLGL2* inhibited the ovarian cancer cell migration and invasion abilities in vitro without affecting cell proliferation. LLGL2-overexpressing mice had fewer metastatic implant foci than the controls in vivo. The insignificant difference might result from the different impact of LLGL2 upon the cell proliferation, as well as the effect of microenvironment.

Epithelial–mesenchymal transition (EMT) is highly related to tumor cell migration, invasion, and metastasis [29,30]. Several EMT-related transcription factors, such as ZEB1, snail, and LOXL2, can inhibit *LLGL2* expression [16,32,33,34]. However, our results showed that overexpression or knockdown of *LLGL2* alone did not cause significant changes in the expression of EMT markers (CDH1 and VIM) or cell junction-related genes (OCLN and TJP1). Our results suggested that although the EMT process causes the expression and functional inhibition of polarity complexes, the loss of the apical–basal polarity, and the establishment of the anterior–posterior polarity in epithelial cells [4,31], overexpression or knockdown of *LLGL2* alone did not alter the cellular EMT state, nor did it reverse the EMT process. *LLGL2* may inhibit the migration and invasion of ovarian cancer cells in an EMT-independent manner. The prefunctional enrichment results suggested that actin assembly might play an important role. We further verified whether *LLGL2* could regulate the cytoskeleton. There were significant decreases in the numbers of intracellular stress fibers and filamentous pseudopods in the *LLGL2* overexpression group compared with those in the empty vector control group. In contrast, the knockdown of *LLGL2* resulted in an increase in intracellular stress fiber formation and in increases in the number and length of filamentous pseudopods compared with those in the negative control. To explore possible *LLGL2*-interacting molecules that regulate the cytoskeleton in ovarian cancer, we performed immunoprecipitation with a monoclonal antibody specific for LLGL2 and subsequent mass spectrometry analysis to identify possible interacting molecules. ACTN1, which is an actin-crosslinking protein, caught our attention. α-Actinin-1 crosslinks actin filaments into actin bundles and networks. Our results demonstrated that LLGL2 interacted with ACTN1 but did not change the expression level of *ACTN1*. α-Actinin-1 was enriched at one side of the cell in *LLGL2*-overexpressing cells while being primarily dispersed in the cytoplasm and the nucleus in the vector control cells. Overall, our results suggested that *LLGL2* interacted with *ACTN1* and affected the affinity of *ACTN1* and actin, thereby leading to cytoskeletal remodeling of ovarian cancer cells and to the inhibition of their invasion and metastasis.

## 5. Conclusions

*LLGL2* is upregulated in ovarian cancer tissues but is negatively correlated with malignant progression and a poor prognosis of ovarian cancer. We uncovered that *LLGL2* altered the intracellular localization and function of *ACTN1* by interacting with *ACTN1* and regulated cytoskeleton remodeling to inhibit the invasion and metastasis of ovarian cancer.

## Data Availability

The original data presented in the study are included in the article/Supplementary Materials. Further inquiries can be directed to the corresponding authors.

## Supplementary Materials

The following supporting information can be downloaded: https://www.mdpi.com/article/10.3390/cancers15245880/s1, Table S1: clinical characteristics of the two groups in GSE65986, Table S2: list of genes enriched in GO: 0030036 and GO: 0030866, Figure S1: overall survival (OS) curves of *PLCL1*, *EPB41L1*, *HIP1R*, *ADD2*, *ACTN4*, *BIK*, *CDK2AP2*, *NCKAP1*, *CLSTN1*, and *CIT* in ovarian cancer patients (CSIOVDB), Figure S2: LLGL1 had no consistently significant differences from *LLGL2*, Figure S3: *LLGL2* did not affect cell proliferation, Figure S4: *LLGL2* inhibited ovarian cancer cell metastasis independently of EMT.
