# Supplementary material for: LLGL2 Inhibits Ovarian Cancer Metastasis by Regulating Cytoskeleton Remodeling via ACTN1
Source: Cancers (Basel). 2023 Dec 18;15(24):5880. doi: 10.3390/cancers15245880 (PMC10742334; doi:10.3390/cancers15245880)

Full unedited gel for Figure 3A

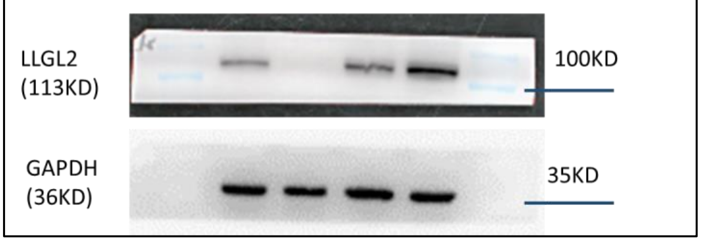

Full unedited gel for Figure 3C

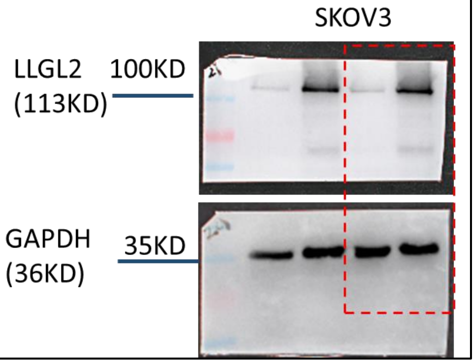

Full unedited gel for Figure 3B

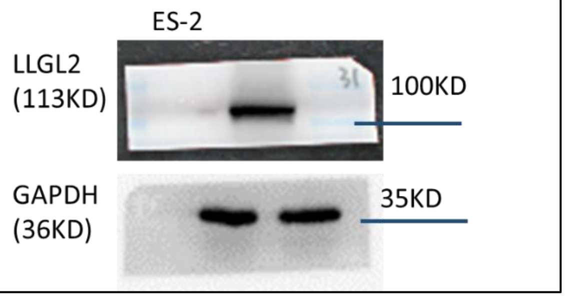

Full unedited gel for Figure 3D

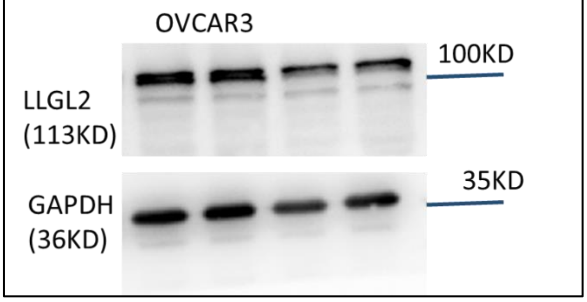

Full unedited gel for Figure 5D

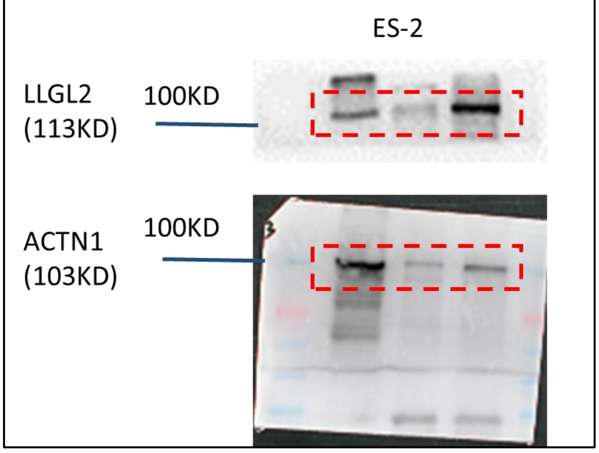

Full unedited gel for Figure 5E

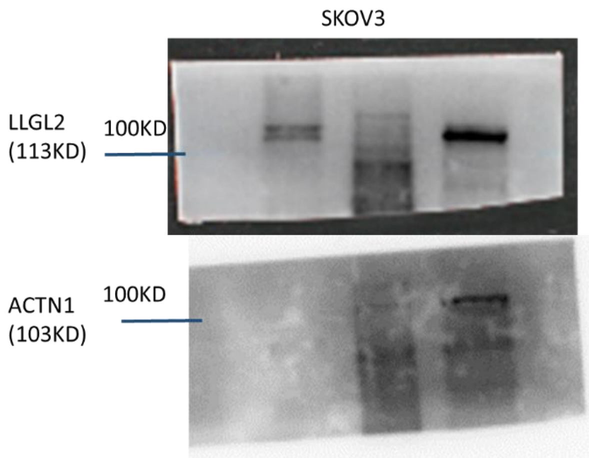

Full unedited gel for Figure 5H

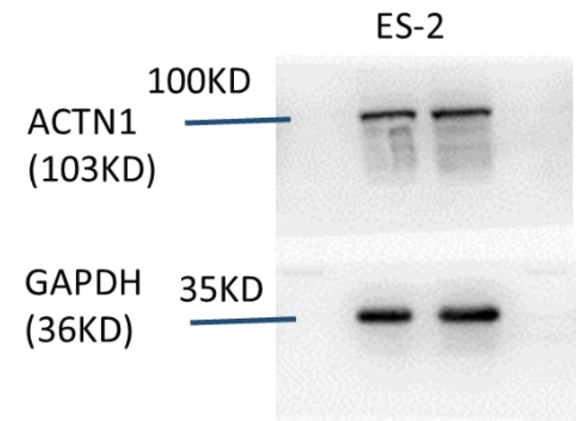

Full unedited gel for Figure 5I

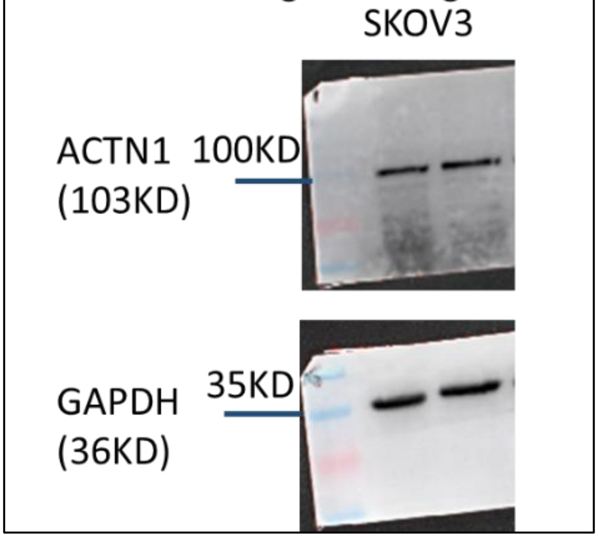

Supplement: Supplementary file 1 [file cancers-15-05880-s001.zip › cancers-2698058-original-images.pdf]
