# Supplementary material for: LLGL2 Inhibits Ovarian Cancer Metastasis by Regulating Cytoskeleton Remodeling via ACTN1
Source: Cancers (Basel). 2023 Dec 18;15(24):5880. doi: 10.3390/cancers15245880 (PMC10742334; doi:10.3390/cancers15245880)
Supplement: Supplementary file 1 [file cancers-15-05880-s001.zip › cancers-2698058-supplementary.pdf]

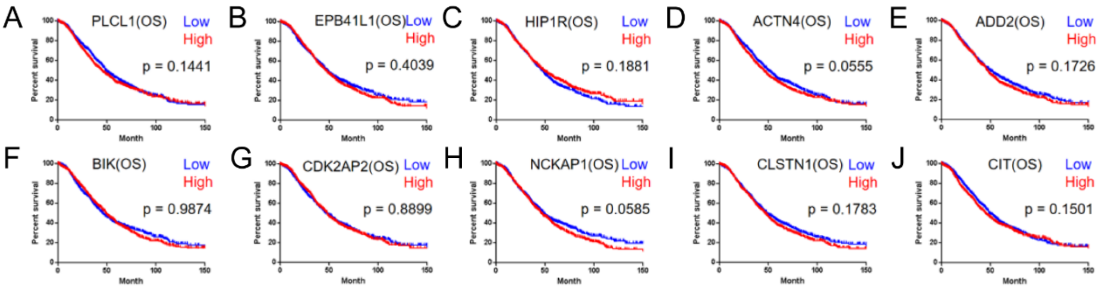

Figure S1 Overall survival (OS) curves of *PLCL1, EPB41L1, HIP1R*, *ADD2*, *ACTN4*, *CDK2AP2*, *NCKAP1*, *CLSTN1* and *CIT* in ovarian cancer patients (CSIOVDB).

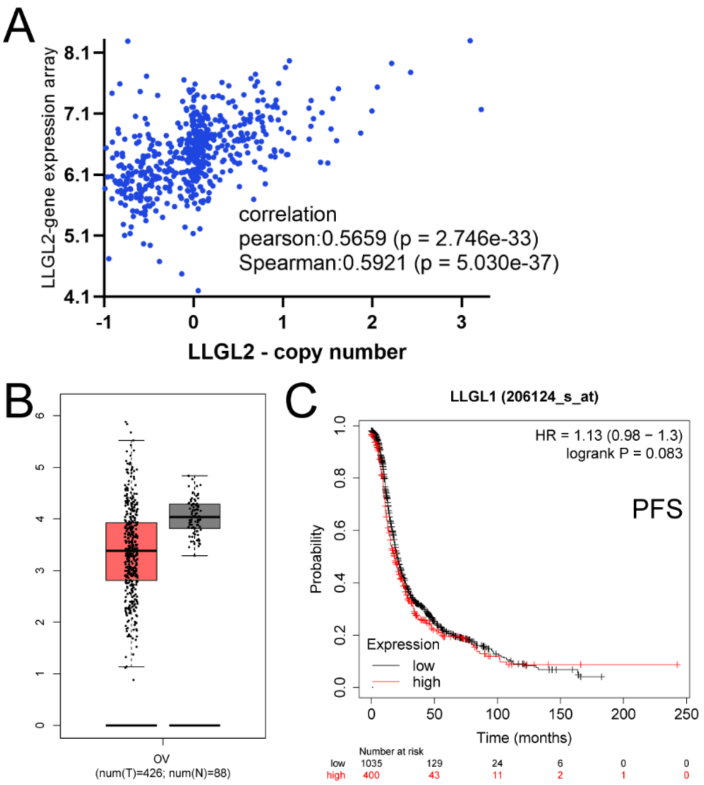

Figure S2. *LLGL1* had no consistently significant differences from LLGL2. (A) Correlation between copy number variation of *LLGL2* and mRNA expression in OV (data from UCSC Xena). (B) *LLGL1* expression in tumor and normal tissues in OV from TCGA. (C) Kaplan–Meier plot of survival of *LLGL1* in patients with ovarian cancer.

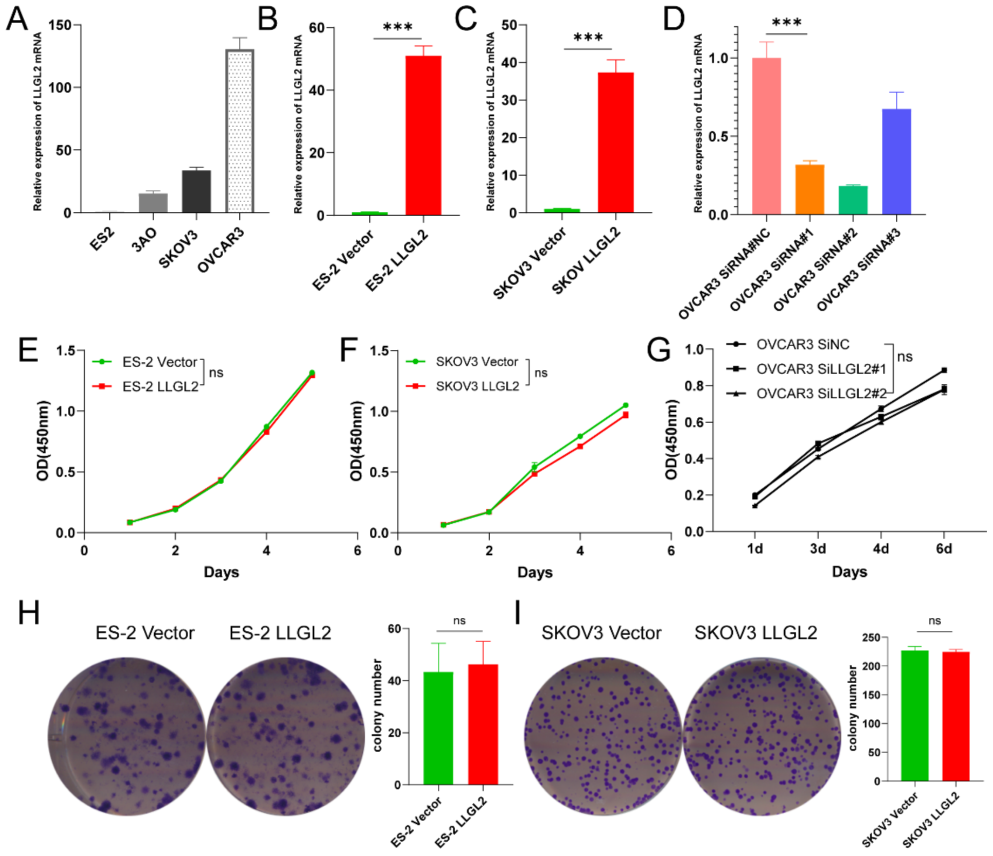

Figure S3. *LLGL2* did not affect cell proliferation. (A)The relative mRNA expression levels of *LLGL2* in ovarian cancer cell lines. (B) The efficiency of LLGL2 overexpression in OCCC cells ES-2 by lentiviral was tested by qPCR. (C)The efficiency of LLGL2 overexpression in SOC cells SKOV3 by lentiviral was tested by qPCR. (D)The efficiency of LLGL2 silencing in SOC cells OVCAR3 by small interfering RNA was tested by qPCR. (E-F) Proliferation curve of control (Vector) and LLGL2-overexpressing ES-2 cells and SKOV3 cells. (G) Proliferation curve of control (NC) and LLGL2- knockdown OVCAR3 cells. (H-I) Colony formation of control (Vector) and LLGL2-overexpressing ES-2 cells and SKOV3 cells.

*P<0.05, **P<0.01, ***P<0.001

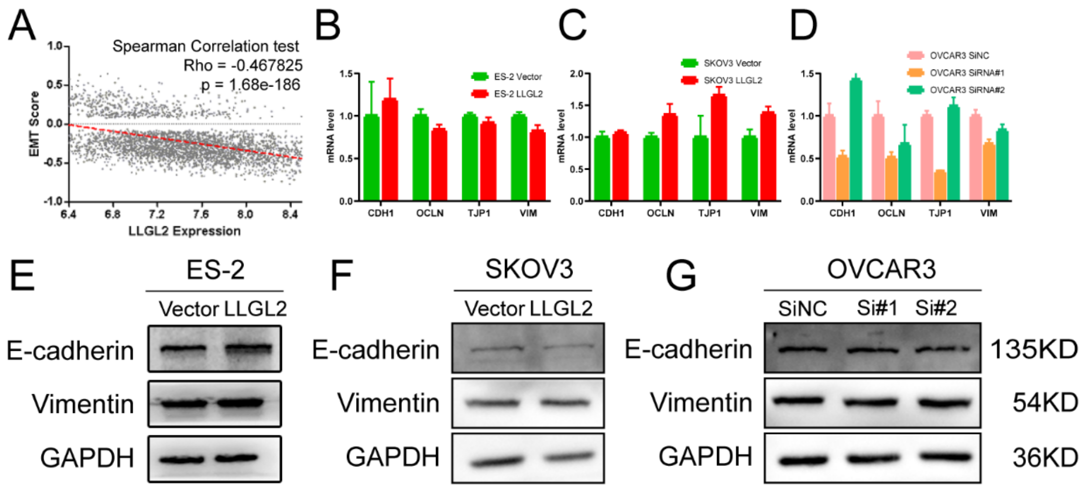

Figure S4 *LLGL2* inhibited ovarian cancer cell metastasis independently of EMT. (A)LLGL2 was negatively correlated with the EMT score from CSIOVDB. (B-G) EMT marker expression in indicated cells was detected by qPCR (B-D) and Western blot (E-G).

Supplemental Tables

Table S1. Clinical characteristics of the two groups in GSE65986.

P： ID	age	histology	pfs	prognosis	event	Stage	stage	group
GSM1612098	57	Clear	57	NED	0	1c	early	good
GSM1612099	59	Clear	52	NED	0	1a	early	good
GSM1612103	67	Clear	49	NED	0	1a	early	good
GSM1612104	37	Clear	48	NED	0	1c	early	good
GSM1612119	74	Clear	11	AWD	1	3c	advanced	bad
GSM1612117	55	Clear	6	DOD	1	1c	early	bad
GSM1612116	80	Clear	5	AWD	1	4	advanced	bad
GSM1612118	64	Clear	2	DOD	1	3c	advanced	bad

Table S2. List of genes enriched in GO: 0030036 and GO: 0030866

Gene	Description	Expression
ADD2	adducin 2	Up-regulation
ACTN4	actinin alpha 4	Up-regulation
HIP1R	huntingtin interacting protein 1 related	Down-regulation
EPB41L1	erythrocyte membrane protein band 4.1 like 1	Up-regulation
CLSTN1	calsyntenin 1	Up-regulation
PLCL1	phospholipase C like 1 (inactive)	Down-regulation
BIK	BCL2 interacting killer	Up-regulation
LLGL2	LLGL scribble cell polarity complex component 2	Up-regulation
CIT	citron rho-interacting serine/threonine kinase	Up-regulation
NCKAP1	NCK associated protein 1	Up-regulation
CDK2AP2	cyclin dependent kinase 2 associated protein 2	Up-regulation
